# Perceptions and experiences of individuals at-risk of rheumatoid arthritis (RA) knowing about their risk of developing RA and being offered preventive treatment: systematic review and thematic synthesis of qualitative studies

**DOI:** 10.1136/annrheumdis-2021-221160

**Published:** 2021-11-08

**Authors:** Heidi J Siddle, Lara S Chapman, Kulveer Mankia, Codruța Zăbălan, Marios Kouloumas, Karim Raza, Marie Falahee, Joel Kerry, Andreas Kerschbaumer, Daniel Aletaha, Paul Emery, Suzanne H Richards

**Affiliations:** 1 Leeds Institute of Rheumatic and Musculoskeletal Medicine, University of Leeds, Leeds, UK; 2 NIHR Leeds Biomedical Research Centre, Leeds, UK; 3 Romanian League against Rheumatism, Bucharest, Romania; 4 Federation of Patient Associations of Cyprus, Nikosia, Cyprus; 5 Rheumatology Research Group, Institute of Inflammation and Ageing, College of Medical and Dental Sciences, University of Birmingham, Birmingham, UK; 6 Department of Rheumatology, Sandwell and West Birmingham NHS Trust, Birmingham, UK; 7 Library and Information Service, Leeds Teaching Hospitals NHS Trust, Leeds, UK; 8 Department of Rheumatology, Medical University of Vienna, Vienna, Austria; 9 Leeds Institute of Health Sciences, University of Leeds, Leeds, UK

**Keywords:** qualitative research, arthritis, rheumatoid, Psychology

## Abstract

**Objectives:**

There is increasing interest in identifying individuals at-risk of rheumatoid arthritis (RA) and initiating early treatment to prevent or delay the onset of arthritis. We aimed to describe the perceptions and experiences of at-risk individuals and to inform the conduct of clinical trials and studies, and clinical practice.

**Methods:**

A systematic review and thematic synthesis of qualitative studies was conducted. Two review authors independently screened studies for inclusion, appraised their methodological quality using the Critical Appraisal Skills Programme checklist and assessed confidence in the findings using the Grading of Recommendations Assessment, Development and Evaluation–Confidence in Evidence from Reviews of Qualitative Research approach.

**Results:**

Seven studies involving 115 individuals at-risk of developing RA were included. Three major themes (seven subthemes) were identified: understanding the risk of developing RA (knowledge of RA and identification of potential risk factors); preventive interventions to reduce the risk of developing RA (understanding the value and role of preventive interventions, and engagement with preventive interventions); and perceptions of predictive testing for RA (benefits of predictive testing, decision to undertake predictive testing and concerns about predictive testing). Moderate confidence in most review findings was evident.

**Conclusion:**

While there are clear benefits in informing individuals at-risk of RA about their risk following predictive testing and offering preventive treatment, there are potential barriers to engagement, intensified by the burden of uncertainty. Identification of the optimum approaches for presenting risk information, including the risks and benefits of engaging with preventive interventions, is urgently needed to support individuals at-risk of RA in their decision making.

**PROSPERO registration number:**

CRD42021236034.

## Introduction

Rheumatoid arthritis (RA) is a chronic inflammatory arthritis with a profound impact on quality of life and function. Identifying individuals with early RA and early initiation of treatment has shown to be effective at reducing the long-term damage associated with the erosive and persisting nature of the disease.^1–3^ As well as improved clinical outcomes, early identification has also been associated with improved health-related quality of life and work ability.^4^

Research regarding the early diagnosis of RA has resulted in prediction models and international classification criteria,^5–9^ and there is an increasing focus on the efficacy of preventive medication in the preclinical phase to prevent or delay the progression of RA.^10–14^ However, there is limited insight into how at-risk individuals understand their own risk and on their views regarding predictive testing and engaging with preventive interventions, including lifestyle changes and medication. A metasynthesis of qualitative studies exploring the perceptions of predictive testing for those at-risk of developing a chronic inflammatory disease has previously been conducted.^15^ This review identified patients’ concerns about confidentiality, lack of motivation for change, poor clinician–patient communication and impact of the test result on emotional well-being as perceived barriers to predictive testing. A patient-centred approach throughout the testing process, including accessible information and support for patients to engage in risk-reducing health behaviours, was recommended. However, this review did not identify any participants at-risk of developing RA; the majority of studies included participants at-risk of diabetes and cardiovascular disease. These diseases are more prevalent than RA, routinely screened for in healthcare, and their risk factors and implications are better understood by the general public, with fewer misconceptions.^16^

In order to identify, recruit, offer intervention and monitor individuals at-risk of developing RA, it is imperative to appreciate potential barriers and facilitators to individuals’ understanding of their risk and their motivations for engaging in clinical trials and studies. This is particularly important, given the current uncertainty about whether or when those who have been identified as at-risk of RA will actually develop clinical arthritis.

The aim of the current study was to synthesise qualitative studies exploring the perceptions and experiences of individuals at-risk of developing RA, to inform the conduct of clinical trials and studies, and clinical practice.

## Methods

We followed the Enhancing Transparency of Reporting the Synthesis of Qualitative Research framework in reporting this review.^17^

### Inclusion criteria

We included qualitative studies in which the authors undertook interviews or focus groups with adults (>18 years) at-risk of developing RA to explore the perceptions or experiences of being informed of this risk or being offered preventive treatment. At-risk populations eligible for inclusion were (1) asymptomatic at-risk individuals, which includes first-degree relatives (FDRs) of people with RA and indigenous North Americans; (2) at-risk individuals with musculoskeletal symptoms without clinical arthritis; and (3) at-risk individuals with early clinical arthritis, which includes patients with palindromic rheumatism and undifferentiated arthritis.^18^ We included full articles in the English language that were published in peer-reviewed journals. Conference abstracts were excluded. Mixed-methods studies reporting quantitative and qualitative data were only eligible for inclusion if the qualitative data could be extracted separately. Studies including participants other than at-risk individuals (eg, healthcare professionals or patients with a diagnosis of RA) were included only if the data on eligible at-risk participants could be separated from the data on ineligible participants.

### Search strategy

A literature search was performed using MEDLINE, Embase, PubMed and the Cochrane Central Register of Controlled Trials from inception to April 2021. The search strategy was conducted with guidance from a health librarian (JK) and is included within online supplemental file 4. An extensive manual search of reference lists and related citations of relevant articles was also conducted, followed by forward citation tracking using Scopus. Finally, we held discussions about the literature with experts in this field, including authors of included articles, to minimise the likelihood of overlooking any additional relevant material.

10.1136/annrheumdis-2021-221160.supp4 Supplementary data

### Study selection and data extraction

All activities were undertaken by researchers trained in qualitative methods (HJS and LSC) and under the supervision of an experienced qualitative methodologist (SHR). Studies retrieved from the searches were recorded on a central database. After excluding duplicate articles, two review authors (HJS and LSC) independently screened all titles and abstracts. Full texts of the studies identified as being potentially eligible for inclusion were then independently assessed against the inclusion and exclusion criteria by the two review authors. The following data were extracted electronically from eligible articles by one review author (LSC) using a standardised data collection form in Microsoft Excel (Microsoft Office Professional Plus 2016): study details (lead author and year of publication); participants (at-risk population, sample size and demographic characteristics); setting (country); data collection method (interview or focus group); recruitment technique; patient involvement; and data analysis method. All data within the results section of each study were extracted, including themes, subthemes, supporting verbatim quotations and the authors’ interpretations of the data. Any disputes were settled by discussion between the two review authors or resolved through further discussion with two other members of the review team (SHR and KM) where necessary.

### Quality assessment

Two review authors (HJS and LSC) independently assessed the quality of each included study using the Critical Appraisal Skills Programme (CASP) checklist for qualitative studies.^19^ Any discrepancies were discussed until consensus was reached or resolved in further discussion with SHR and KM. The CASP checklist consists of 10 items, and each item includes multiple signalling questions to help users interpret the item (29 signalling questions in total). A summary table detailing the frequency of responses to each signalling question was constructed.^20^ The CASP checklist has no scoring matrix; therefore, a narrative summary of the quality of the individual included studies is provided.

### Data synthesis and analysis

We used the method of thematic synthesis described by Thomas and Harden to identify and develop themes from our included articles.^21^ All extracted data from the Results section of each study were considered in the synthesis. Two review authors (HJS and LSC) read each article multiple times to achieve immersion, then independently performed line-by-line coding of the data to search for concepts. Following comparisons of common convergent and divergent concepts within and across studies, codes were organised into related areas to construct descriptive themes and subthemes. This was achieved through an iterative process of translating concepts from one study to another by adding coded text to existing concepts and creating new concepts when deemed necessary. The preliminary coding framework was discussed with a third author (SHR). Descriptive themes were then inductively analysed further to construct analytical themes, to ‘go beyond’ the findings reported in our included studies and generate additional understanding relating to our research question.^21^ Both review authors then reread each included article to ensure themes were represented in the primary data, and illustrative verbatim quotations were incorporated. The proposed descriptive and analytical themes were subsequently presented, discussed and finalised with the entire review team, including two patient research partners (CZ and MK).

### Assessment of confidence in the review findings (Grading of Recommendations Assessment, Development and Evaluation–Confidence in Evidence from Reviews of Qualitative Research (GRADE-CERQual))

Two review authors (HJS and LSC) independently assessed the confidence in each individual review finding using the GRADE-CERQual approach.^22^ Four components were considered to formulate an overall assessment of confidence in each synthesised qualitative finding: methodological limitations (using CASP), coherence of data, adequacy of data and relevance of the studies.^23–27^ Both review authors then independently judged overall confidence in each review finding as high, moderate, low or very low. Full definitions of each GRADE-CERQual component and confidence ratings are presented in online supplemental file 2. Disagreements in confidence ratings were resolved via discussion or through inclusion of a third author (SHR).

10.1136/annrheumdis-2021-221160.supp2 Supplementary data

### Patient and public involvement

International patient research partners (CZ and MK) were engaged throughout each stage, including during the development of the review question and interpretation of the results, through attendance at study meetings and contributions to ongoing discussions about the findings. Our patient research partners reviewed each manuscript draft; their feedback resulted in changes to the presentation of source data and thematic schema, and to the overall structure of the results.

## Results

### Study selection

**Figure 1:**
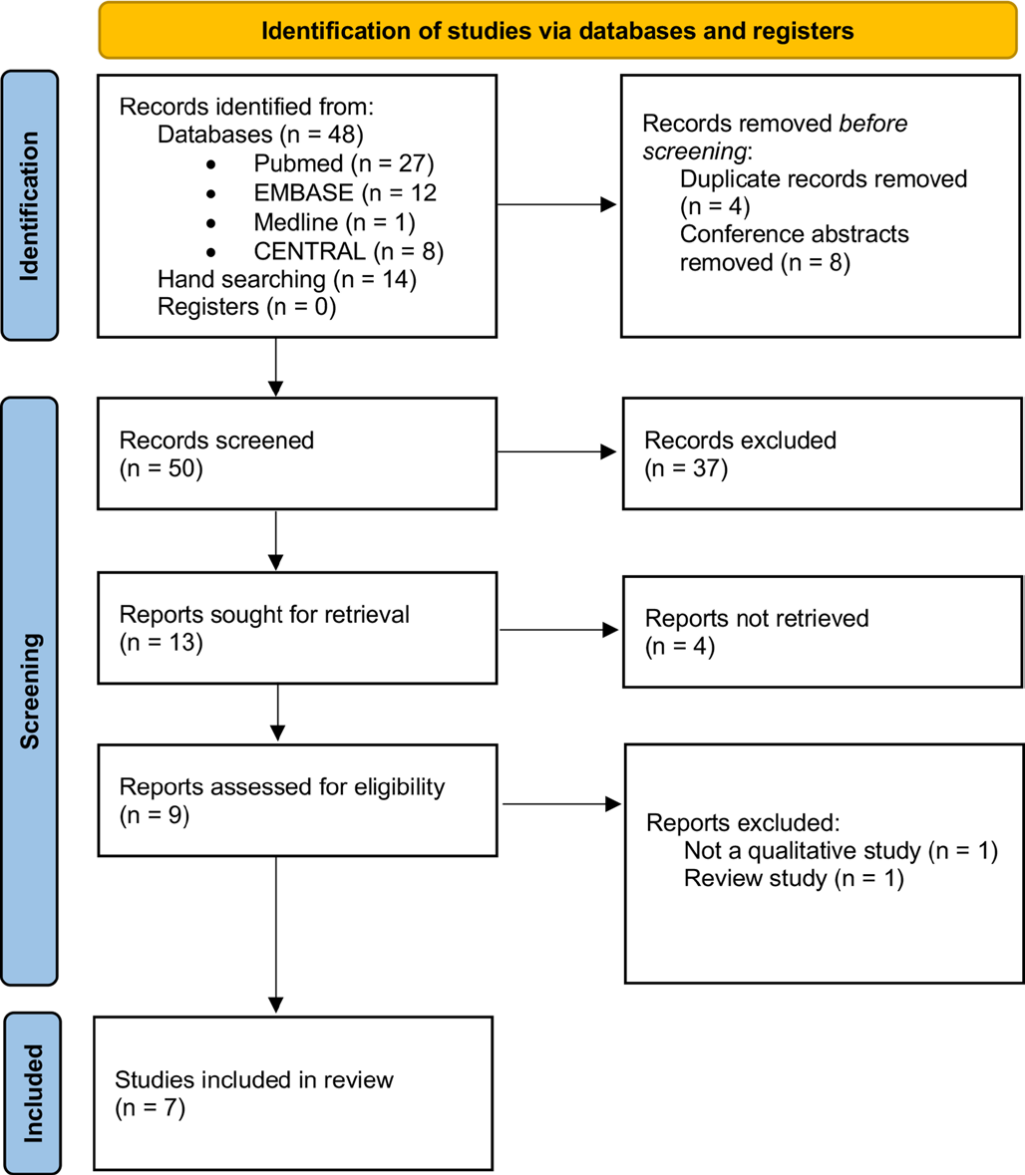
PRISMA flow diagram (adapted from Page *et al*).^44^ PRISMA, Preferred Reporting Items for Systematic Reviews and Meta-Analyses.

In total, the searches yielded 62 records, of which 9 were retrieved for full-text screening. Seven studies representing six data sets met our inclusion criteria. The full selection process is presented in a Preferred Reporting Items for Systematic Reviews and Meta-Analyses 2020 flow diagram (figure 1). Studies were conducted in the UK, Austria, Germany, Switzerland, the Netherlands and Canada. The sample includes 115 individuals at-risk of RA. Table 1 provides an overview of the study characteristics and participant demographics. Four studies^28–31^ used individual semistructured interviews, whereas three studies^32–34^ used focus groups.

**Table 1:** Study characteristics

Study ID	At-risk population	Sample size	Age	Female	Ethnicity	Setting (country)	Data collection method	Recruitment technique	Patient involvement	Analysis
Mosor *et al* 2020^28^	24 ACPA/RF positive with arthralgia (9 FH RA); 10 asymptomatic ACPA/RF positive (1 FH RA)	34	Mean (SD): symptomatic 48.6 (14.4), asymptomatic 61.7 (9.6), total 52.4 (14.4); range 18–81	26	Not reported	Rheumatology centres (15 Austria, 15 Germany, 4 UK)	Semistructured interviews	Individuals were either referred for testing because of symptoms or had a predictive test for RA as part of an extended medical check-up.	English interview guide co-developed with patient research partners; results reviewed by patient research partners.	Thematic
Munro *et al* 2018^32^	FDRs	5	Mean (SD) 29.4 (12.4)	Not reported	Not reported	Arthritis Consumer Experts/Joint Health Group and Arthritis Research Canada Arthritis Patient Advisory Board (Canada)	Focus group	Marketing and communications lists of the Arthritis Consumer Experts/Joint Health group and Arthritis Research Canada Arthritis Patient Advisory Board mailing lists, or snowballing sampling through the patient participants	Semistructured interview guide informed by consultation with a patient partner	Framework
Newsum *et al* 2016^33^	4 with CSA (1 ACPA and RF positive; 1 FH RA; 2 seronegative)	4	Mean 37.5, range 24–54	4	Not reported	Secondary care (Netherlands)	Focus group	Randomly selected individuals with arthralgia of <1 year of hand or foot joints without clinical arthritis at physical examination and an increased risk of developing clinical arthritis according to the rheumatologists were approached by telephone and asked to participate in the focus group discussion.	Not reported	IPA
Novotny *et al* 2013^29^	FDRs; 4 siblings, 14 parents; 2 parents/grandparents	20	Mean (SD) 45 (12), range 21–78	18	Not reported	Hospital rheumatology department (Switzerland)	Semistructured interviews	Announcements in the press to invite FDRs of patients with RA to participate in a cohort study; to encourage participation, the main biomarkers predicting RA were assayed free of charge.	Not reported	Thematic
Simons et al* 2018^30^	FDRs; 6 siblings, 26 parents, 2 siblings/parents	34	Mean (SD) 39 (10.8); Range 21–67	26	32 White, 2 Asian	Secondary care (24 UK, 3 Germany, 7 Austria)	Semi-structured interviews	Patients with RA were approached during their routine secondary care clinic appointments and asked to consider contacting a FDR about participating in an interview study about risk and predictive testing for RA.	Interview schedule was informed by consultation with patient research partners; patient research partners blind coded three transcripts; coding framework discussed with patient research partners.	Thematic
Stack *et al* 2016* 31	FDRs; 6 siblings, 26 parents, 2 siblings/parents	34	Mean 39, range 21–67	26	32 white, 2 Asian	Secondary care (24 UK, 3 Germany, 7 Austria)	Semistructured interviews	Patients with RA were approached during their routine secondary care clinic appointments and were given a letter to pass on to an FDR of their choosing inviting them to participate in an interview about risk and predictive testing for RA.	Patient research partners reviewed and redrafted the interview schedule and blind coded three transcripts; discussion of the coding framework took place between researchers and patient research partners.	Thematic
van Boheemen *et al* 2021^34^	ACPA or ACPA and RF positive with arthralgia and no history of clinical arthritis	18	Mean (SD) 59 (9)	10	Not reported	Not reported	Focus group	Participants of a prevention trial or individuals who declined trial participation but consented to be contacted	Not reported	Thematic

*Same data set.

ACPA, anticitrullinated protein antibody; CCP, cyclic citrullinated peptide; CSA, clinically suspect arthralgia; FDR, first-degree relative; FH, family history; IPA, interpretive phenomenological analysis; RA, rheumatoid arthritis; RF, rheumatoid factor.

### Quality appraisal

The frequency of responses (‘yes’ or ‘no’) to each signalling question in the CASP checklist is detailed in online supplemental file 3. Strengths observed in all studies included clearly stated objectives, appropriate methodology and design, clearly stated and justified data collection methods, and confirmation of ethical approval. The following limitations were identified in at least five studies: no discussions around recruitment (eg, why some people chose not to take part)^29–32 34^; no justification for data collection setting^28 29 32–34^; no discussion of the issues raised by the study^28 29 32–34^; no critical examination of the researchers’ own role, potential bias and influence during the formulation of the research question and data collection^28–34^; and no critical examination of the researcher’s own role, potential bias and influence during analysis and selection of data for presentation.^28–32 34^

10.1136/annrheumdis-2021-221160.supp3 Supplementary data

### Synthesis of qualitative studies

**Figure 2:**
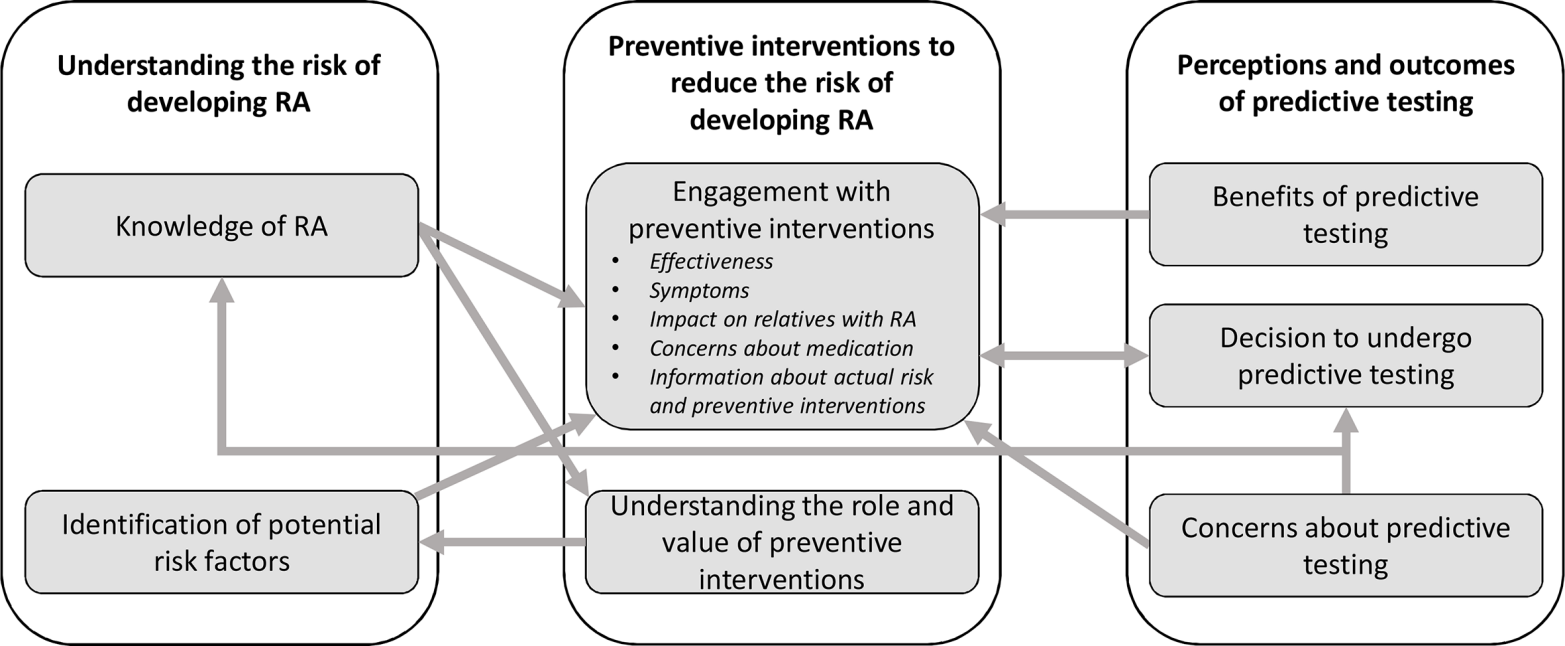
Thematic schema. RA, rheumatoid arthritis.

Our thematic synthesis identified seven descriptive themes describing the perceptions and experiences of study participants. These were organised into three major analytical themes: understanding the risk of developing RA (theme 1), preventive interventions to reduce the risk of developing RA (theme 2) and perceptions of predictive testing for RA (theme 3). Illustrative quotes for each major theme are presented in tables 2–4, and conceptual links among themes are displayed in figure 2.

**Table 2:** Theme 1 illustrative quotes

Theme 1: understanding the risk of developing RA	
Descriptive theme	Illustrative quotes
Knowledge of RA	
Individuals at-risk of RA have gained knowledge of RA through experiencing symptoms or witnessed the impact of RA on their relatives.	‘However, I do notice that I want to avoid certain situations. For instance, sometimes I put off visitors because I know they won’t understand I am in pain. Or because they don’t take into account that I have to stand up on my feet quite often. Then I prefer to say ‘Well, not today, thank you,’ instead of joining them for an outing’.^33^ ‘She [family member] had a life and then once the disease came and took it from her, she didn’t [anything] anymore. She couldn’t do things’.^32^
Individuals at-risk of RA identified a need for more knowledge about RA and risk factors.	‘Up until now I have never thought about it, what that would be like, whether it might happen’.^31^ ‘And I’ve heard theories, everything from it [RA] skips generations to it’s immediate, to you know it only affects the women in one side of the family. I’ve heard a whole bunch of different crazy different things’.^32^
Identification of potential risk factors	
Individuals at-risk of RA perceived that certain factors increase the risk of developing RA.	‘Yeah, I looked it [information about RA] up online, and yes, then you see how bad it can get, and I think, well, I’m not that far along yet’.^34^ ‘So I know it’s blood-related…I think if it was your cousin or your aunt there’d be a slim chance…being direct blood-related, I would class myself as, or think of myself that I am at a higher risk than most’.^31^ ‘I think it probably half depend on what kind of person you are, I know for my sister she was much more worried than I was only because she’s a lot older than me and she’s overweight and she saw that as kind of, like without reading the letters I could figure she was going to get it more than me’.^30^

RA, rheumatoid arthritis.

**Table 3:** Theme 2 illustrative quotes

Theme 2: preventive interventions to reduce the risk of developing RA	
Descriptive theme	Illustrative quotes
Understanding the role and value of preventive interventions	
Individuals at-risk of RA acknowledged that preventive interventions have a role in modifying risk.	‘I think drugs would be involved, drugs that are less strong than those used to treat the disease’.^29^ ‘ifestyle changes, I’m up for any kind really, yeah. Healthy eating and exercise, although I can’t do a lot but I do try and do as much as I can’.^30^
Engagement with preventive interventions	
Individuals at-risk of RA identified that engagement with a preventive intervention would be influenced by its effectiveness in reducing risk.	‘I’ve got to take a medication for how long, the rest of my life? … It’s a big commitment when the odds of developing the disease is still fairly high if I’ve got a 50% risk of still developing it, whereas if you tell me, ‘Well, actually, if you take it and based on what we can tell you about your predictability factors, your odds of developing the disease are gonna be down to 5%,’ then I might consider it’.^30^
Having symptoms would make individuals at-risk of RA more willing to consider preventive interventions.	‘Well, changing lifestyle means changing diet, difficult, because changing your diet, abstaining from certain food that you like to eat, means reducing your quality of life. I personally don’t agree with that, I’m definitely not going on a diet because of a disease I don’t have at the moment! But I certainly would if I had any symptoms’.^28^ ‘The chance that I would do it would increase hand over hand if I had severe pain’.^34^
Seeing the impact of RA on a relative would make individuals at-risk of RA more willing to consider preventive interventions.	‘RA is in my family unfortunately. My mother, my grandmother, they’re both gone (…). And the fact that I participate in the medication trial is just like, yes, I’ve seen what RA can do’.^34^
Individuals at-risk of RA had concerns about taking preventive medication.	‘I prefer a drug that doesn’t affect the immune system(…)drugs can make us more vulnerable to infections’.^29^ ‘You know, I went to Europe last year with my wife. We were gone for, you know, half a year. Now if I wasn’t able to do that because I had to go to a specific doctor twice a week to get this thing, no thanks. I’m good’.^32^
Individuals at-risk of RA highlighted a need for more information about their actual risk and preventive interventions before engaging.	‘Only under the condition that a person would receive the necessary information to be able to decide whether to take a preventive medicine’.^28^ ‘From where it would be coming from, Dr.— was like, ‘Hey, you know, there’s this treatment. You know, I know how badly it effects your mother. I think that you are possibly at-risk for having it,’ and he suggested it to me, I would definitely take a look at it’.^32^

RA, rheumatoid arthritis.

**Table 4:** Theme 3 illustrative quotes

Theme 3: perceptions of predictive testing for RA	
Descriptive theme	Illustrative quotes
Benefits of predictive testing	
Individuals at-risk of RA perceived predictive testing as useful.	‘I think that with kind of information, I’d be more keen to, sort of, sort out what I needed to do to try and prevent that becoming a problem. If I could take some sort of medication to…head it off before it became a big problem’. 31 Yes, I have pain in the joints regularly and that’s why it was interesting to me to find out the results. I think it was just confirmation that my feeling wasn’t just made up of thin air’.^28^
Decision to undergo predictive testing	
Presence of symptoms, perceived effectiveness and understanding of the impact of disease affect individuals’ decision to undergo predictive testing	‘If there were perhaps a treatment that were extremely preventive and very effective at lessening the risk of developing such a disease, I absolutely would take the test because that to me leads to something that is preventive. That leaves me being able to take some action’.^31^ ‘It’s like looking into a crystal ball [of a fortune teller] and saying to you, “Oh, you could potentially get rheumatoid arthritis.” And then, always, I have images of people in my mind who have deformities and disabilities’.^28^
Concerns about predictive testing	
Individuals at-risk of RA had concerns about predictive testing.	‘Because if told me—it’s only how likely, it’s not a, ‘You will develop it,’ and it doesn’t tell you when you will develop it. So I think if somebody said to me, ‘There’s this test out there and it’ll tell you whether you might develop it,’ I wouldn’t want it, because you could just live your life in fear and never actually develop it. So unless it was 100% guaranteed, and somebody could say, ‘You will develop it within this time frame,’ I don’t wanna [want to] spend the next 30 years worrying about something, when I could be enjoying those 30 years. So, no, I’d probably—it depends on the exact details of the test’.^31^ ‘Statistics like 1 out of 10 really don’t mean a thing to me. The way I reason is, I am not 1 out of 10. That’s how I feel about it, it won’t be me’.^33^

RA, rheumatoid arthritis.

### Theme 1: understanding the risk of developing RA

Within theme 1, two descriptive subthemes were identified relating to understanding the risk of developing RA: knowledge of RA and identification of potential risk factors.

#### Knowledge of RA

Many participants had witnessed the severity and impact of RA on their relatives.^29–34^ Some participants expressed concerns about developing RA, perceiving that it would be painful and unpredictable, restricting daily living.^31–33^ Participants with symptoms revealed experiences of unpredictable pain and fatigue, with negative consequences such as reduced ability to participate in hobbies and social outings, and perceived that these issues would worsen if they developed RA.^33^

Some participants recognised they lacked knowledge about RA and their risk as a relative.^31 32 34^ Participants felt they needed more information about RA and its related risk factors, particularly to inform their decision making around preventive interventions and undergoing predictive testing.^28 30–32 34^

#### Identification of potential risk factors

In four studies, participants directly or indirectly identified known risk factors for developing RA, including diet, being overweight, smoking, family history and being female.^30–33^ Other participants identified what they considered to be risk factors, such as sports participation and ageing, which have not been identified as predictors of developing RA in empirical research.

### Theme 2: preventive interventions to reduce the risk of developing RA

Within theme 2, two descriptive themes related to preventive interventions to reduce the risk of developing RA: understanding the value and role of preventive interventions, and engagement with preventive interventions.

#### Understanding the value and role of preventive interventions

Five studies discussed the role of preventive interventions in reducing the risk of developing RA.^28–30 32 34^ Preventive interventions identified by participants included medications, lifestyle changes, screening and alternative medicines (eg, herbal treatments). Some participants suggested that preventive medication would be similar to that received by their relatives with RA,^30 32^ while others thought preventive medication would be ‘less strong’.^29^ Specific lifestyle changes considered by participants included healthy eating, increased exercise and smoking cessation.^28 30 34^ While most studies focused on the role of treatments in preventing the development of RA, participants in one study acknowledged that preventive interventions might at best delay the onset of RA rather than stop it altogether.^29^

#### Engagement with preventive interventions

Some participants expressed willingness to engage with preventive interventions, including through participation in research.^29 30^ However, most participants indicated that their perceived engagement with preventive interventions would depend on a balance of certain factors; primarily the effectiveness of preventive interventions in reducing risk, their experience of symptoms, seeing the impact of RA on a relative, adverse effects of preventive medication and information provided by health professionals.

Some participants expressed that the effectiveness of an intervention in reducing their risk of developing RA would affect their decision to engage.^30 32 34^ Participants confirmed that the presence of RA symptoms would make them more likely to engage with preventive interventions.^28–30 34^ Understanding of the impact of RA also affected participants’ perceived or actual engagement with preventive interventions, with some expressing a willingness to consider medication or participate in a clinical trial involving medication, to prevent symptoms of the disease that they had witnessed in relatives.^29 30 34^

Many participants had concerns about preventive medication specifically.^28–30 34^ In some cases, these concerns were based on negative attitudes towards taking medications in general. Other participants had concerns about the side effects of preventive medication, particularly if they currently felt healthy.^29 30 32 34^ These included physical side effects, such as infections and liver damage, and psychological side effects. In some cases, these concerns were based on participants’ experiences of seeing relatives take medication for RA. Two studies also identified concerns relating to the administration of medication^29 32^; participants indicated a preference for tablets over injections due to the perceived impact of having regular injections on their lives, for example, limitations in travelling.

Participants identified the need to weigh up the pros and cons of engaging with a preventive intervention. This involved taking into consideration their risk of developing RA against the effectiveness and adverse effects of preventive medication or the perceived negative consequences of making lifestyle changes.^29 30 34^ Participants highlighted a need for more information about their actual risk before engaging with preventive interventions. Additionally, participants recognised a need for more information about preventive medication, including adverse effects and mode of administration, to inform their decision making.^28 30 32 34^ Some participants suggested they would be more likely to take medication or participate in a clinical trial involving preventive medication, if a trusted health professional recommended it.^32 34^ One study, undertaken in Canada, also acknowledged cost as a potential factor that could affect engagement with preventive medication.^32^

Some participants acknowledged they would be more willing to make lifestyle changes, undergo screening or take alternative medicines than take preventive medication.^28 30 32 34^ Participants in one study had already adopted healthy behaviours in an attempt to deal with their arthralgia, including dietary changes, mindfulness and yoga,^33^ while other participants acknowledged their attempts to live as healthily as possible regardless of their risk of developing RA.^30^

### Theme 3: perceptions of predictive testing for developing RA

Three descriptive subthemes themes contributed to the main theme of perceptions of predictive testing for developing RA: benefits of predictive testing, decision to undergo predictive testing and concerns about predictive testing.

#### Benefits of predictive testing

Some participants perceived predictive testing as useful to clarify their symptoms or risk status,^28 31 33^ prepare mentally and physically for the future,^31^ or to contribute to research to ultimately help other people.^28 31^ Other participants recognised that confirmation of their risk of developing RA would allow them to be proactive about their health, prompting them to monitor early symptoms and report changes to a health professional, make lifestyle changes or take preventive medication.^28 30–32^

#### Decision to undergo predictive testing

Participants’ decision to undergo predictive testing was influenced by presence of symptoms. One study identified that symptomatic participants were more likely to undergo predictive testing than asymptomatic participants.^28^ However, another study reported that a small number of symptomatic participants were fearful of clarification and had not sought further advice.^31^ The perceived effectiveness of preventive interventions may also influence participants’ decision to undergo predictive testing, prompting them to take action.^32^ For some participants, the decision to undergo predictive testing would be influenced by the ability of a test to provide a definitive result and a confirmed timeline for developing RA, rather than a probability.^31^ Some participants suggested they would take the opinion of a trusted health professional into consideration and might be more likely to undergo predictive testing if it was recommended.^32^

#### Concerns about predictive testing

Participants in two studies described feeling fearful and anxious about the outcome of predictive testing,^31 32^ perceiving that confirmation of risk status would reduce their ability to enjoy life.^31^ These concerns were compounded by uncertainty around whether or not the disease would actually develop.

Other participants had concerns about the accuracy of the test,^28 31 32^ the impact of a false-positive result^31 32^ and the potential for predictive testing to trigger further invasive tests, such as biopsies.^28^ Some participants described how their relatives with RA also had concerns about predictive testing, especially as both parties may not have considered participants’ susceptibility of developing the condition prior to taking part in research.^31^

Two studies discussed communication of test results.^28 31^ Some participants had concerns about how their test results would be communicated, based on their previous negative experiences of receiving other test results, and suggested that information should be understandable without the use of medical terms and with accompanying examples to enhance comprehension.^28^ Another study revealed that participants felt they were unable to interpret prognostic information in terms of probabilities of their symptoms progressing to RA.^33^ Participants recognised a need for support from health professionals throughout the predictive testing process during both the delivery of test results and in the at-risk stage.^28 31^

Participants in two studies had already undergone predictive testing.^28 31^ Reactions to the test result varied; most asymptomatic participants reported feeling calm, while symptomatic participants described feeling anxious, shocked, worried about the future (with regards to the potential impact on working, for example), and had difficulties in discussing the outcome with others.^28^

### Assessment of confidence in the review findings (GRADE-CERQual)

We had moderate confidence in most of the review findings (table 5; a detailed GRADE-CERQual Qualitative Evidence Profile is also presented in the online supplemental file 1). This was due primarily to concerns regarding methodological limitations, adequacy of the data (due to the limited number of studies meeting our inclusion criteria) and relevance of each contributing study to the review question (given the absence of studies of participants with early clinical arthritis, one study failing to report its setting and all of the remaining studies from high-income countries, with none having recruited from primary care). We had low confidence in two findings: engagement with a preventive intervention would be influenced by its effectiveness in reducing risk, and the presence of symptoms, perceived effectiveness and understanding of the impact of disease affect individuals’ decisions to undergo predictive testing.

10.1136/annrheumdis-2021-221160.supp1 Supplementary data

**Table 5:** GRADE-CERQual summary of review findings

Summary of review finding	Studies contributing to the finding	GRADE-CERQual assessment of confidence in the evidence
Individuals at-risk of RA have gained knowledge of RA through experiencing symptoms or witnessing the impact of RA on their relatives	29–34	Moderate confidence
Individuals at-risk of RA identified a need for more knowledge about RA and risk factors.	30–32 34	High confidence
Individuals at-risk of RA perceived that certain factors increase the risk of developing RA.	29–32	Moderate confidence
Individuals at-risk of RA acknowledged that preventive interventions have a role in modifying risk.	28–30 32 34	Moderate confidence
Individuals at-risk of RA identified that engagement with a preventive intervention would be influenced by its effectiveness in reducing risk.	30 32 34	Low confidence
Having symptoms would make individuals at-risk of RA more willing to consider preventive interventions.	28–30 34	Moderate confidence
Seeing the impact of RA on a relative would make individuals at-risk of RA more willing to consider preventive interventions.	29 30 34	Moderate confidence
Individuals at-risk of RA had concerns about taking preventive medication.	28–30 32 34	Moderate confidence
Individuals at-risk of RA highlighted a need for more information about their actual risk and preventive interventions before engaging.	28–30 32 34	Moderate confidence
Individuals at-risk of RA perceived predictive testing as useful.	28 30–33	Moderate confidence
Presence of symptoms, perceived effectiveness and understanding of the impact of disease affect individuals’ decision to undergo predictive testing.	28 31	Low confidence
Individuals at-risk of RA had concerns about predictive testing.	28 31–33	Moderate confidence

GRADE-CERQual, Grading of Recommendations Assessment, Development and Evaluation–Confidence in Evidence from Reviews of Qualitative Research; RA, rheumatoid arthritis.

## Discussion

This review informs our understanding of the factors that may influence the willingness of individuals at-risk of RA to undertake predictive testing and engage with preventive interventions. We specifically focused on the perceptions and experiences of individuals at-risk of RA, rather than those of health professionals or patients with RA.

Although individuals discussed potential risk factors for developing RA, including smoking and increased weight, in this review, they did not identify certain modifiable risk factors for developing the condition, such as the contribution of poor dental hygiene and periodontal disease.^35^ The potential gap in knowledge among individuals at-risk of RA regarding poor dental health as a risk factor for developing the disease is also in concordance with the previous literature.^36^ Our review indicates that understanding the risk of developing RA is underpinned by the individual’s knowledge of both RA and the risk factors for developing the disease. In a study assessing knowledge of RA risk factors among asymptomatic FDRs, baseline knowledge of this risk factor was low, but increased significantly following personalised RA educational intervention.^36^ The effectiveness of providing personalised risk information to FDRs to calculate disease risk^37^ and increase motivation to improve RA risk-related behaviours,^38^ has previously been demonstrated.

While our synthesis indicated that participants were willing to make lifestyle changes to prevent or delay the onset of RA, we identified some misconceptions relating to risk factors and subsequent lifestyle changes. For example, some participants within our review incorrectly identified ageing as a risk factor. This suggests there is confusion between RA and osteoarthritis, in concurrence with a previous qualitative exploration of illness perceptions of RA in the general public.^16^ Our findings reveal that the decision to engage with preventive medication is multifactorial and links closely with knowledge of risk and the resulting disease. Our review identified that at-risk individuals would be more willing to make lifestyle changes than take preventive medication, in concordance with the previous literature.^1 14^ This is in contrast to a previous survey involving rheumatologists, where the majority were unlikely to advise lifestyle changes and were more willing than at-risk individuals to start preventive medication, regardless of side effects.^14^

The personal burden of living with RA, including illness uncertainty, has been well established.^39^ Our review indicated that individuals at-risk of RA may have concerns about predictive testing and finding out their risk status because of this perceived burden. Illness uncertainty has been identified as a cognitive stressor that impacts on treatment adherence.^40^ Patients with RA have expressed uncertainty about symptoms and prognosis, treatment effectiveness and toxicity, and potential consequences of the disease on their lives.^41^ Our findings suggest that individuals at-risk of RA have similar experiences but must also manage the additional uncertainty of future disease progression. A common finding across all themes was individuals’ need for further information, which is accurate and personalised to the individual at-risk of RA, to inform decision making around preventive interventions and predictive testing. However, our review has established that while information provided by health professionals can be influential, many individuals at-risk of RA also draw on their experiences of relatives living with RA to inform their decision making.

Perceptions of predictive testing among individuals at-risk of RA identified in our review are similar to those identified in a previous meta-synthesis of qualitative studies involving participants with other chronic inflammatory diseases (diabetes, cardiovascular disease and inflammatory bowel disease).^15^ This meta-synthesis identified the benefits of predictive testing to motivate lifestyle changes, but also revealed the potential negative emotional impact of testing. In congruence with our synthesis, previous surveys conducted with individuals at-risk of RA^14 42^ and spondyloarthropathy^43^ highlighted participants’ concerns about preventive medication, particularly with regard to side effects. For example, in one such study, willingness to take preventive medication decreased by approximately half with the possibility of mild side effects.^43^ Synonymous with our review findings, previous studies have also revealed that the decision to engage with preventive interventions depends on the effectiveness of these treatments,^14 42^ the opinions of trusted healthcare professionals,^42^ individuals’ perceptions of how severe the disease is^43^ and when their risk of developing the disease is increased.^14 43^ However, in contrast to our findings, one previous study found that mode of drug administration did not influence at-risk individuals’ decisions to take preventive treatment.^43^ Further understanding of how the delivery of preventive medication affects people’s perceptions and decision making is required.

To our knowledge, this is the first study that synthesises existing qualitative literature on the perceptions and experiences of individuals at-risk of developing RA. We systematically assessed and coded all relevant data using established and prespecified methodology. At least two review authors were involved in study selection, data extraction, CASP assessments and coding of data, reducing the potential for errors, and we formally assessed our confidence in each review finding using the GRADE-CERQual approach. Our review findings should be considered in light of some limitations. First, only seven studies met our inclusion criteria, and two of these studies were from the same data set. We carried out an extensive search of the literature to ensure no relevant articles were missed. The small number of included studies reflects the lack of qualitative studies undertaken in this evolving area of RA research, highlighting the need for further studies in this area. Although our review included 115 participants from six countries, we recognise that some of the original studies were conducted in their native language and then translated into English for publication. This has the potential for cultural meanings to be modified in our thematic synthesis and theme development. Second, overall confidence in each finding was hindered somewhat by methodological limitations of the studies, particularly lack of reporting around non-participation characteristics and rates. It is possible that individuals who were more engaged with the idea of predictive testing and preventive interventions were willing to participate. Future research in this area should aim to minimise the impact of this limitation, for example, by asking individuals who decline participation to detail their reasons for this decision. Third, the participants included within our review were either individuals with musculoskeletal symptoms but without clinical arthritis or FDRs of individuals with RA recruited through secondary care; therefore, our findings provide limited insights into the perceptions and experiences of other individuals at-risk of developing the condition, such as other asymptomatic at-risk individuals (eg, indigenous North Americans who are at increased genetic risk) and at-risk individuals with early clinical arthritis, including patients with palindromic rheumatism and undifferentiated arthritis.^18^ We acknowledge that the themes we have identified might potentially differ between the different groups of individuals at-risk included within this review (eg, in terms of their knowledge of RA), as well as groups of at-risk individuals not represented in study samples. Additionally, while our search strategy included the term ‘inflammatory arthritis’, no qualitative studies relating to patients at-risk of inflammatory arthritides other than RA were found. Therefore, our review specifically focused on RA and our findings may not be transferable to other forms of inflammatory arthritis, although surveys have demonstrated similar perspectives of risk and preventive interventions among individuals at-risk of spondyloarthropathy.^14 43^ Finally, only two studies explicitly reported the ethnic background of participants, and all studies were conducted in high-income countries; therefore, findings may not be transferable to individuals from ethnic minority groups or to different healthcare settings. Other factors, such as gender, cultural background, socioeconomic status and health literacy levels, may also influence the decision to undertake predictive testing and engage with preventive interventions. Future research should focus on these gaps.

Several implications arise from our review. Our findings suggest that while there are benefits in informing individuals at-risk of developing RA about their risk and offering preventive treatment, there are potential barriers to engagement among these individuals. We propose that individuals’ knowledge about their risk of developing RA may inform their decision to engage with preventive interventions, including medication and lifestyle changes. We recommend that individuals be informed about their risk of developing RA using a personalised approach, ensuring they understand risk factors, their personal risk and how to reduce this risk, and addressing any misconceptions. A previous randomised controlled trial has demonstrated that personalised educational tools can support communication of risk in this population,^37^ but prognostic information based on risk percentages may not be considered as useful to individuals at-risk of RA.^33^ We propose that communication should be tailored to the individual, with accessible, patient-understandable information on the impact of preventive interventions provided. Participants’ educational status and literacy levels should also be considered, as these may affect their decisions and needs. Information should include the nature and likelihood of immediate and long-term physical and psychological side effects, medication administration and the anticipated effectiveness of the intervention. Fundamental to this tailored communication is wider exploration of the concerns individuals may have based on their own experiences. Further support from trusted health professionals should be available for at-risk individuals, particularly taking into consideration the potential negative emotional impact of testing and the additional burden of uncertainty that testing may produce. This will become increasingly important in clinical practice as the focus of rheumatology care shifts to prevention of disease in individuals at-risk of RA as opposed to intervention in early RA. Future studies should establish the optimum approaches for conveying the risk of developing RA to at-risk individuals, and determine how at-risk individuals assess risk versus benefit when deciding whether to engage with preventive interventions. Our recommendations primarily aim to inform the conduct of future clinical trials and observational studies, but are also applicable to broader clinical practice.
