# Supplementary material for: Perceptions and experiences of individuals at-risk of rheumatoid arthritis (RA) knowing about their risk of developing RA and being offered preventive treatment: systematic review and thematic synthesis of qualitative studies
Source: Ann Rheum Dis. 2021 Nov 8;81(2):159–68. doi: 10.1136/annrheumdis-2021-221160 (PMC8762008; doi:10.1136/annrheumdis-2021-221160)
Supplement: Supplementary data [file annrheumdis-2021-221160supp004.pdf]

Supplementary material. Search strategy.

1. "rheumatoid arthritis" OR "rheumatoid nodulosis" OR "caplan's syndrome" OR "still's disease" OR "inflammatory arthritis" OR "felty syndrome" OR RA OR "rheumatoid vasculitis").ti,ab

2. "ARTHRITIS, RHEUMATOID"/

3. (1 OR 2)

4. (risk OR "risk factors" OR "relative risk").ti,ab

5. RISK/ OR "RISK FACTORS"/ OR “RELATIVE RISK”/

6. (4 OR 5)

7. ("patient adherence" OR "patient information" OR "patient compliance" OR "patient education").ti,ab

8. "PATIENT COMPLIANCE"/ OR "PATIENT ACCEPTANCE OF HEALTH CARE"/

9. "PATIENT EDUCATION AS TOPIC"/ OR “PATIENT EDUCATION”/

10. “PATIENT INFORMATION”/

11. (7 OR 8 OR 9 OR 10)

12. "PREVENTIVE MEDICINE"/

13. (prevent* OR "preventative medicine").ti,ab

14. (11 OR 12)

15. (3 AND 6 AND 11 AND 14)
