# Supplementary material for: Perceptions and experiences of individuals at-risk of rheumatoid arthritis (RA) knowing about their risk of developing RA and being offered preventive treatment: systematic review and thematic synthesis of qualitative studies
Source: Ann Rheum Dis. 2021 Nov 8;81(2):159–68. doi: 10.1136/annrheumdis-2021-221160 (PMC8762008; doi:10.1136/annrheumdis-2021-221160)
Supplement: Supplementary data [file annrheumdis-2021-221160supp002.pdf]

Supplementary table. GRADE-CERQUAL confidence rating definitions.

**GRADE-CERQual confidence rating**	**Definition**
High	Highly likely that the review finding is a reasonable representation of the phenomenon of interest.
Moderate	Likely that the review finding is a reasonable representation of the phenomenon of interest.
Low	Possible that the review finding is a reasonable representation of the phenomenon of interest.
Very low	Unclear whether the review finding is a reasonable representation of the phenomenon of interest
