# Supplementary material for: Perceptions and experiences of individuals at-risk of rheumatoid arthritis (RA) knowing about their risk of developing RA and being offered preventive treatment: systematic review and thematic synthesis of qualitative studies
Source: Ann Rheum Dis. 2021 Nov 8;81(2):159–68. doi: 10.1136/annrheumdis-2021-221160 (PMC8762008; doi:10.1136/annrheumdis-2021-221160)
Supplement: Supplementary data [file annrheumdis-2021-221160supp003.pdf]

Supplementary table. GRADE-CERQual Qualitative Evidence Profile.

Summary of review finding	Studies contributing to the finding	Methodological limitations	Coherence	Adequacy	Relevance	CERQual assessment of confidence in the evidence	Explanation of CERQual assessment
**Theme 1: Understanding the risk of developing RA**							
Individuals at-risk of RA have gained knowledge of RA through experiencing symptoms or witnessing the impact of RA on their relatives	[29-34]	Moderate concerns: two studies [Simons, Stack] with minor concerns (unclear recruitment, no critical examination of own role in designing and conducting the study, no critical examination of own role during analysis); three studies [Munro, van Boheemen, Newsum] with moderate concerns (unclear recruitment, no justification of data collection setting, no critical examination of own role in designing and conducting or the study, no discussion of issues raised/effects of study on participants, no explanation of how the data presented were selected from the original sample, no critical examination of own role during analysis, no identification of new areas where research is necessary); one study with serious concerns [Novotny] (no discussions around recruitment, no justification for data collection setting, no critical examination of own role in	No or very minor concerns.	No or very minor concerns.	Moderate concerns: four studies were with FDRS but only one of these included NA FDRs; two studies were of at-risk individuals with symptoms; no studies of at-risk individuals with early clinical arthritis; all studies were recruited through secondary care; five studies were from high-income countries, whilst one study didn't report the setting.	Moderate confidence	Moderate concerns about methodological limitations and relevance

		designing and conducting the study, insufficient ethical considerations, no discussion of issues raised/effects of study on participants, no critical examination of own role during analysis, inadequate discussion of evidence, no discussion of credibility of findings, no discussion of study's contribution, no identification of new areas where research is necessary).					
Individuals at-risk of RA identified a need for more knowledge about RA and risk factors	[30-32, 34]	Minor concerns: two studies [Simons, Stack] with minor concerns (unclear recruitment, no critical examination of own role in designing and conducting the study, no critical examination of own role during analysis); two studies [Munro, van Boheemen] with moderate concerns (unclear recruitment, no justification of data collection setting, no critical examination of own role in designing and conducting the study, no discussion of issues raised/effects of study on participants), no critical examination of own role during analysis, no identification of new areas where research is necessary.<br><br>Recruitment, data coll justified, issues raised by study, critical ex own role in	No or very minor concerns.	No or very minor concerns.	Moderate concerns: three studies were with FDRS but only one of these included NA FDRs; only one study of at-risk individuals with symptoms; no studies of individuals with early clinical arthritis; three studies were recruited through secondary care; three studies were from high-income countries, whilst one study did not report the setting.	High confidence	Moderate concerns about methodological limitations and relevance.

		designing, new areas where research is necessary.					
Individuals at-risk of RA perceived that certain factors increase the risk of developing RA	[29-32]	Moderate concerns: two studies [Simons, Stack] with minor concerns (unclear recruitment, no critical examination of own role in designing and conducting the study, no critical examination of own role during analysis); one study with moderate concerns [Munro] (unclear recruitment, no justification of data collection setting, no critical examination of own role in designing and conducting the study, no critical examination of own role during analysis, no discussion of issues raised/effects of study on participants); one study with serious concerns [Novotny] (no discussions around recruitment, no justification for data collection setting, no critical examination of own role in designing and conducting the study, insufficient ethical considerations, no discussion of issues raised/effects of study on participants, no critical examination of own role during analysis, inadequate discussion of evidence, no discussion of credibility of findings, no discussion of study's contribution, no identification of new areas where research is necessary).	No or very minor concerns.	Minor concerns: four studies contributed to this finding – two had rich supporting data and two had superficial supporting datal, but this is a superficial descriptive finding (identification of risk factors only).	Moderate concerns: four studies were with FDRS but only one of these included NA FDRs; no representation of at-risk individuals with symptoms or individuals with early clinical arthritis; all studies were recruited through secondary care; all studies were from high-income countries.	Moderate confidence	Moderate concerns about methodological limitations and relevance, minor concerns about adequacy.
**Theme 2: Preventive interventions to reduce the risk of developing RA**							

Individuals at-risk of RA acknowledged that preventive interventions have a role in modifying risk	[28-30, 32, 34]	Moderate concerns: one study [Simons] with minor concerns (unclear recruitment, no critical examination of own role in designing and conducting the study, no critical examination of own role during analysis); one study [Novotny] with serious concerns (no discussions around recruitment, no justification for data collection setting, no critical examination of own role in designing and conducting the study, insufficient ethical considerations, no discussion of issues raised/effects of study on participants, no critical examination of own role during analysis, inadequate discussion of evidence, no discussion of credibility of findings, no discussion of study's contribution, no identification of new areas where research is necessary); three studies with moderate concerns [Munro, Mosor] (unclear recruitment, no justification of data collection setting, no critical examination of own role in designing and conducting the study, no discussion of issues raised/effects of study on participants), no critical examination of own role during analysis, no identification of new areas where research is necessary).	No or very minor concerns.	No or very minor concerns.	Moderate concerns: four studies were with FDRS but only one of these included NA FDRs; two studies of at-risk individuals with symptoms; no studies of individuals with early clinical arthritis; all studies were recruited through secondary care; four studies were from high-income countries, whilst one study did not report the setting.	Moderate confidence	Moderate concerns about methodological limitations and relevance.

Individuals at-risk of RA identified that engagement with a preventive intervention would be influenced by its effectiveness in reducing risk	[30, 32, 34]	Moderate concerns: one study [Simons] with minor concerns (unclear recruitment, no critical examination of own role in designing and conducting the study, no critical examination of own role during analysis); two studies with moderate concerns [Munro, van Boheeemen] (unclear recruitment, no justification of data collection setting, no critical examination of own role in designing and conducting the study, no discussion of issues raised/effects of study on participants), no critical examination of own role during analysis, no identification of new areas where research is necessary).	Minor concerns: some participants are willing to engage regardless of potential treatment effectiveness, and some individuals are more likely to engage than others – especially with regards to lifestyle changes.	Moderate concerns: three studies contributed but one study provided very superficial data (one quote).	Moderate concerns: two studies were with FDRS but only one of these included NA FDRs; only one study of at-risk individuals with symptoms; no studies of at-risk individuals with early clinical arthritis; all studies were recruited through secondary care; two studies were from high-income countries, whilst one study did not report the setting.	Low confidence	Moderate concerns about methodological limitations, adequacy and relevance.
Having symptoms would make individuals at-risk of RA more willing to consider preventive interventions	[28-30, 34]	Moderate concerns: one study [Simons] with minor concerns (unclear recruitment, no critical examination of own role in designing and conducting the study, no critical examination of own role during analysis); two studies [Mosor, van Boheemen] with moderate concerns (no justification of data collection setting, no critical examination of own role in designing and conducting the study, no discussion of issues raised/effects of study on participants, no critical examination of	No or very minor concerns.	Moderate concerns: four studies contributed and three provided superficial data only, but this is a simple, descriptive finding.	Moderate concerns: two studies were with FDRs, but neither included representation of NA FDRs; two studies of at-risk individuals with symptoms; no studies of early clinical arthritis; all studies were recruited through secondary care; three studies were from high-income countries, whilst one study did not report the setting.	Moderate confidence	Moderate concerns about methodological limitations, adequacy and relevance.

		own role during analysis, no identification of new areas where research is necessary); one study [Novotny] with serious concerns (no discussions around recruitment, no justification for data collection setting, no critical examination of own role in designing and conducting the study, insufficient ethical considerations, no discussion of issues raised/effects of study on participants, no critical examination of own role during analysis, inadequate discussion of evidence, no discussion of credibility of findings, no discussion of study's contribution, no identification of new areas where research is necessary).					
Seeing the impact of RA on a relative would make individuals at-risk of RA more willing to consider preventive interventions	[29, 30, 34]	Moderate concerns: one study [Simons] with minor concerns (unclear recruitment, no critical examination of own role in designing and conducting the study, no critical examination of own role during analysis); one study with moderate concerns [van Boheemen] (no justification of data collection setting, no critical examination of own role in designing and conducting the study, no discussion of issues raised/effects of study on participants, no critical examination of own role during analysis, no	No or very minor concerns	No or very minor concerns.	Moderate concerns: two studies were with FDRS but none included NA FDRs; only one study of at-risk individuals with symptoms; no studies of individuals with early clinical arthritis; all studies were recruited through secondary care; two studies were from high-income countries, whilst one study did not report the setting.	Moderate confidence	Moderate concerns about methodological limitations and relevance.

		identification of new areas where research is necessary); one study [Novotny] with serious concerns (no discussions around recruitment, no justification for data collection setting, no critical examination of own role in designing and conducting the study, insufficient ethical considerations, no discussion of issues raised/effects of study on participants, no critical examination of own role during analysis, inadequate discussion of evidence, no discussion of credibility of findings, no discussion of study's contribution, no identification of new areas where research is necessary).					
Individuals at-risk of RA had concerns about taking preventive medication	[28-30, 32, 34]	Moderate concerns: one study [Simons] with minor concerns (unclear recruitment, no critical examination of own role in designing and conducting the study, no critical examination of own role during analysis); three studies with moderate concerns [Munro, Mosor, van Boheemen] (unclear recruitment, no justification of data collection setting, no critical examination of own role in designing and conducting the study, no discussion of issues raised/effects of study on participants, no critical examination of own role during analysis, no	No or very minor concerns.	No or very minor concerns	Moderate concerns: three studies were with FDRS but only one of these included NA FDRs; two studies of at-risk individuals with symptoms; no studies of individuals with early clinical arthritis; four studies were recruited through secondary care; four studies were from high-income countries, whilst one study did not report the setting.	Moderate confidence	Moderate concerns about methodological limitations and relevance.

		identification of new areas where research is necessary); one study [Novotny] with serious concerns (no discussions around recruitment, no justification for data collection setting, no critical examination of own role in designing and conducting the study, insufficient ethical considerations, no discussion of issues raised/effects of study on participants, no critical examination of own role during analysis, inadequate discussion of evidence, no discussion of credibility of findings, no discussion of study's contribution, no identification of new areas where research is necessary.					
Individuals at-risk of RA highlighted a need for more information about their actual risk and preventive interventions before engaging	[28-30, 32, 34]	Moderate concerns: one study [Simons] with minor concerns (unclear recruitment, no critical examination of own role in designing and conducting the study, no critical examination of own role during analysis); three studies [Mosor, Munro, van Boheemen] with moderate concerns (unclear recruitment, no justification of data collection setting, no critical examination of own role in designing and conducting the study, no discussion of issues raised/effects of study on participants, no critical examination of own role during analysis, no	Minor concerns: some participants are willing to engage with lifestyle changes regardless of their actual risk.	Minor concerns: five studies contributed, three of these provided rich data, but two provided superficial data only.	Moderate concerns: three studies were with FDRs, but only one of these included NA FDRs; two studies of at-risk individuals with symptoms; no studies of early clinical arthritis; four studies were recruited through secondary care; four studies were from high-income countries, whilst one study did not report the setting.	Moderate confidence	Minor concerns about coherence and adequacy, moderate concerns about methodological limitations and relevance.

		identification of new areas where research is necessary); one study [Novotny] with serious concerns (no discussions around recruitment, no justification for data collection setting, no critical examination of own role in designing and conducting the study, insufficient ethical considerations, no discussion of issues raised/effects of study on participants, no critical examination of own role during analysis, inadequate discussion of evidence, no discussion of credibility of findings, no discussion of study's contribution, no identification of new areas where research is necessary).					
**Theme 3: Perceptions and outcomes of predictive testing**							
Individuals at-risk of RA perceived predictive testing as useful	[28, 30-33]	Moderate concerns: two studies [Simons, Stack] with minor concerns (unclear recruitment, no critical examination of own role in designing and conducting the study, no critical examination of own role during analysis); three studies [Mosor, Munro, Newsum] with moderate concerns (unclear recruitment, no justification of data collection setting, no critical examination of own role in designing and conducting the study, no discussion of issues raised/effects of study on	Minor concerns: some contradictory statements on how useful it would be to know risk status.	Minor concerns: five studies contributed to the finding, but one provided superficial data only.	Moderate concerns: three studies were with FDRs, but only one of these included NA FDRs; only two studies of at-risk individuals with symptoms; no studies of early clinical arthritis; all studies were recruited through secondary care; all studies were from high-income countries.	Moderate confidence	Minor concerns about coherence, moderate concerns about methodological limitations and relevance.

		participants, no explanation of how the data presented were selected from the original sample, no critical examination of own role during analysis, no identification of new areas where research is necessary).					
Presence of symptoms, perceived effectiveness, and understanding of the impact of disease affect individuals' decision to undergo predictive testing.	[28, 31]	Minor concerns: one study [Stack] with minor concerns (unclear recruitment, no critical examination of own role in designing and conducting the study, no critical examination of own role during analysis); one study [Mosor] with moderate concerns (no justification of data collection setting, no critical examination of own role in designing and conducting the study, no discussion of issues raised/effects of study on participants, no critical examination of own role during analysis, no identification of new areas where research is necessary).	No or very minor concerns.	Serious concerns: only two studies contributed to the finding, and both had superficial supporting data.	Moderate concerns: only one study with FDRs; no studies of NA FDRs; only one study of at-risk individuals with symptoms; no studies of early clinical arthritis; all studies were recruited through secondary care; all studies were from high-income countries.	Low confidence	Minor concerns about methodological limitations, moderate concerns about relevance, and serious concerns about adequacy.
Individuals at-risk of RA had concerns about predictive testing	[28, 31-33]	Moderate concerns: one study [Stack] with minor concerns (unclear recruitment, no critical examination of own role in designing and conducting the study, no critical examination of own role during analysis); three studies [Mosor, Munro, Newsum] with moderate concerns (unclear recruitment, no justification of data collection setting, no critical	Minor concerns: some contradictory statements on how useful it would be to know risk status.	Minor concerns: four studies contributed to the finding, but one of these provided superficial supporting data only.	Moderate concerns: two studies of FDRs, but only one of these included NA FDRs; only two studies of at-risk individuals with symptoms; no studies of early clinical arthritis; all studies were recruited through secondary care; all studies were from high-income countries.	Moderate confidence	Moderate concerns regarding methodological limitations and relevance of the data.

		examination of own role in designing and conducting the study, no discussion of issues raised/effects of study on participants, no explanation of how the data presented were selected from the original sample, no critical examination of own role during analysis, no identification of new areas where research is necessary).					
