# Supplementary material for: Perceptions and experiences of individuals at-risk of rheumatoid arthritis (RA) knowing about their risk of developing RA and being offered preventive treatment: systematic review and thematic synthesis of qualitative studies
Source: Ann Rheum Dis. 2021 Nov 8;81(2):159–68. doi: 10.1136/annrheumdis-2021-221160 (PMC8762008; doi:10.1136/annrheumdis-2021-221160)
Supplement: Supplementary data [file annrheumdis-2021-221160supp001.pdf]

Supplementary table. GRADE-CERQual component definitions.

**GRADE-CERQual component**	**Definition**
Methodological limitations	The extent to which there are concerns about the design or conduct of the primary studies that contributed evidence to an individual review finding.
Coherence	An assessment of how clear and cogent the fit is between the data from the primary studies and a review finding that synthesises the data.
Adequacy	An overall determination of the degree of richness and the quantity of data supporting a review finding.
Relevance	The extent to which the body of data from the primary studies supporting a review finding is applicable to the context specified in the review question.
